# Impact of guidelines for the management of minor head injury on the utilization and diagnostic yield of CT over two decades, using natural language processing in a large dataset

**DOI:** 10.1007/s00330-018-5954-5

**Published:** 2019-01-14

**Authors:** Ewoud Pons, Kelly A. Foks, Diederik W. J. Dippel, M. G. Myriam Hunink

**Affiliations:** 1 Department of Radiology and Nuclear Medicine, Erasmus University Medical Center Rotterdam, Rotterdam, The Netherlands; 2 Department of Medical Informatics, Erasmus University Medical Center Rotterdam, Rotterdam, The Netherlands; 3 Department of Public Health, Erasmus University Medical Center Rotterdam, PO Box 2040, 3000 CA Rotterdam, The Netherlands; 4 Department of Neurology, Erasmus University Medical Center Rotterdam, Rotterdam, The Netherlands; 5 Department of Epidemiology, Erasmus University Medical Center Rotterdam, Rotterdam, The Netherlands; 6 Center for Health Decision Sciences, Harvard T.H. Chan School of Public Health, Boston, USA

**Keywords:** Craniocerebral trauma, Natural language processing, Data mining, Validation studies, Quality assurance, health care

## Abstract

**Objectives:**

We investigated the impact of clinical guidelines for the management of minor head injury on utilization and diagnostic yield of head CT over two decades.

**Methods:**

Retrospective before-after study using multiple electronic health record data sources. Natural language processing algorithms were developed to rapidly extract indication, Glasgow Coma Scale, and CT outcome from clinical records, creating two datasets: one based on all head injury CTs from 1997 to 2009 (*n* = 9109), for which diagnostic yield of intracranial traumatic findings was calculated. The second dataset (2009–2014) used both CT reports and clinical notes from the emergency department, enabling selection of minor head injury patients (*n* = 4554) and calculation of both CT utilization and diagnostic yield. Additionally, we tested for significant changes in utilization and yield after guideline implementation in 2011, using chi-square statistics and logistic regression.

**Results:**

The yield was initially nearly 60%, but in a decreasing trend dropped below 20% when CT became routinely used for head trauma. Between 2009 and 2014, of 4554 minor head injury patients overall, 85.4% underwent head CT. After guideline implementation in 2011, CT utilization significantly increased from 81.6 to 87.6% (*p* = 7 × 10^−7^), while yield significantly decreased from 12.2 to 9.6% (*p* = 0.029).

**Conclusions:**

The number of CTs performed for head trauma gradually increased over two decades, while the yield decreased. In 2011, despite implementation of a guideline aiming to improve selective use of CT in minor head injury, utilization significantly increased.

**Key Points:**

*• Over two decades, the number of head CTs performed for minor, moderate, and severe head injury gradually increased, while the diagnostic yield for intracranial findings showed a decreasing trend.*

*• Despite the implementation of a guideline in 2011, aiming to improve selective use of CT in minor head injury, utilization significantly increased, while diagnostic yield significantly decreased.*

*• Natural language processing is a valuable tool to monitor the utilization and diagnostic yield of imaging as a potential quality-of-care indicator.*

**Electronic supplementary material:**

The online version of this article (10.1007/s00330-018-5954-5) contains supplementary material, which is available to authorized users.

## Introduction

Non-contrast head CT is routinely used to rule out intracranial complications after (blunt) head trauma [1], but for patients with minor head injury (MHI) or mild traumatic brain injury—Glasgow Coma Scale (GCS) ≥ 13—CT is not always necessary [2]. Intracranial traumatic findings are seen on 7–12% of CTs, although less than 1% of MHI patients require surgery, due to severe complications such as intracranial hematomas [3–6]. Over time, several guidelines have been developed to assess the risk of intracranial complications, using patient characteristics at presentation, such as vomiting or amnesia [3–5, 7]. These guidelines enable selective use of CT, with the goal to avoid unnecessary imaging and therefore reduce utilization. When comparing commonly used guidelines for MHI, the inherent trade-off between sensitivity and specificity with varying cutoff criteria is seen, leading to variation in the number of unnecessary head CTs and missed intracranial findings [8].

The purpose of implementing guidelines is to promote appropriate utilization which leads to safe, cost-effective practice that provides high-quality patient care. In the context of MHI, guidelines commonly reduce utilization. Nevertheless, several studies reported increased utilization of CT after guidelines for selective use were implemented [9, 10], leading to higher costs, longer waiting times, and additional radiation risk [11–13]. After implementation of validated imaging guidelines, it is important to assess their effectiveness in routine clinical practice. Both utilization (i.e., the proportion of patients that undergo imaging) and diagnostic yield (i.e., the proportion of imaging procedures with relevant findings) are important indicators for appropriate use of imaging.

The study purpose is to assess the impact of imaging guidelines for the management of MHI in routine clinical practice, by measuring both utilization and diagnostic yield of CT over two decades. We hypothesized that implementation of improved guidelines for selective use of CT would result in decreased utilization and consequentially also increased diagnostic yield over time.

The large number of clinical records related to MHI in this timeframe made manual review unfeasible. Natural language processing (NLP) can be used to extract structured variables from electronic free text and has been successfully applied to various sources in the electronic health record (EHR) [14], including radiology reports [15]. Therefore, NLP methods were developed to facilitate large dataset analytics of two decades of EHR sources.

## Methods

We performed a retrospective before-after study using multiple EHR data sources from an urban, academic, level 1 trauma center for MHI patients presenting at the emergency department (ED). Part of the data was prospectively collected in the CT in Head Injury Patients (CHIP) study [4].

### Sources and data collection

Several data sources related to MHI were obtained from the EHR, containing information on presentation, diagnostic imaging results, and other potentially relevant clinical outcomes: these sources included clinical notes from neurology, non-contrast head CT reports, neurosurgery registrations, hospitalization records, and various metadata (i.e., age, gender, and time of death for deceased patients).

### NLP development and performance assessment

Four NLP algorithms were developed to:

1. Select acute head trauma cases from clinical notes;
2. Extract GCS score from clinical notes;
3. Select reports ordered for traumatic indication from all head CTs; and
4. Select head CT reports describing any intracranial traumatic finding.

Each NLP algorithm was trained on a set of reference documents, for which two or more clinicians manually labeled all information that should be extracted by NLP. The NLP algorithms for selecting acute head traumas and extracting GCS score were both trained using 500 labeled clinical notes from presentation. Additionally, traumatic indication was manually labeled in 500 head CTs, which were used for training the third NLP algorithm, in order to select traumatic cases from radiology reports directly—before clinical notes were documented electronically in time. Finally, 1934 CT head reports from 2002 to 2003 that had been labeled by our institute during the CHIP study [4] were used to train the fourth NLP algorithm. Therefore, this algorithm selects CT reports with any intracranial traumatic finding (i.e., depressed fracture, subdural hematoma, epidural hematoma, subarachnoid hemorrhage, (non)hemorrhagic contusion, diffuse axonal injury, and intraventricular hemorrhage).

The first NLP algorithm for selecting acute head injuries was optimized for sensitivity to ensure completeness of the data. The fourth algorithm was optimized to balance false positives and false-negative detection of intracranial traumatic findings in a one-to-one ratio, to prevent potential changes in prevalence. During NLP development, 10-fold cross-validation was performed on the labeled reference sets, calculating sensitivity and specificity to measure the performance of all four NLP algorithms.

### Dataset creation and validation

**Fig. 1:**
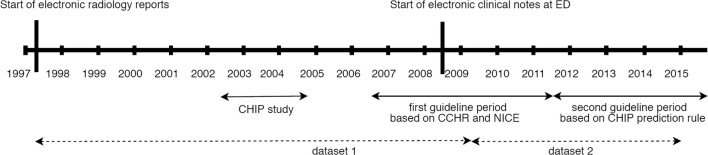
Timeline of guidelines used in the study center and the generated datasets. Dataset 1 contains data extracted from electronic radiology reports between 1997 and 2009 for patients with minor, moderate, and severe head injury; dataset 2 contains data extracted from electronic radiology reports and electronic clinical notes between 2009 and 2014, only for patients with minor head injury. CHIP, CT in head injury patients; CCHR, Canadian CT Head Rule; NICE, National Institute for Health and Care Excellence

After performance evaluation, the four NLP algorithms were applied to all available clinical records (of the type used for training) to extract structured information. Radiology reports were available in digital format from 1997, while clinical notes only existed in the EHR from 2008. Therefore, the extracted variables were grouped into two distinct datasets (Fig. 1).

The first dataset was created by using the NLP algorithms three and four on all radiology reports from 1997 to 2009. This dataset contains minor, moderate, and severe head injury patients, containing CT reports as well as conventional X-ray of the head, which historically had been the first diagnostic test in the workup of head trauma.

The second dataset was created from both CT and clinical reports from 2009 to 2014, using NLP algorithms one, two, and four. Patients with GCS score < 13 were discarded, purposely resulting in a MHI dataset. This dataset also contained all clinical outcomes occurring within 30 days of presentation: hospitalization, neurosurgical intervention, and death. These outcomes were manually checked to ensure no critical lesions were missed by the initial head CT. Furthermore, integrity of this dataset was assessed by inspecting 100 randomly selected entries for completeness and correctness.

### Guideline implementation over time

During the study timeframe, different diagnostic guidelines for MHI were used (Fig. 1). Until 2002, CT was mainly performed in MHI patients after detection of skull fractures on X-ray. From 2002 to 2004, the study center conducted the prospective CHIP study to investigate the risk factors of MHI, during which patients with GCS score of 13–14 and all patients with GCS score of 15 and at least one risk factor underwent CT (Appendix 1).

In 2006, the first local MHI guideline was implemented. This guideline was based on the Canadian CT Head Rule (CCHR) and the National Institute for Clinical Excellence (NICE) guideline to safely reduce CT utilization [3, 5]; CT was indicated in patients with GCS 15 and one risk factor, or a combination of specific risk factors (Appendix 1).

In 2011, the second MHI guideline was implemented based on the CHIP rule [4]. This guideline was developed to achieve a higher reduction in CTs, while identifying all patients with serious complications that require surgery; CT was indicated for patients with one major criterion or two minor criteria (Appendix 1).

The guideline implementation process remained stable over the years; at the study center, guidelines were based on national guidelines and developed in multidisciplinary groups, and regular updates were performed. The guidelines were presented to the involved clinical departments, formally approved by department staff and could easily be consulted online.

### Statistical analysis

We calculated the diagnostic yield for both datasets by taking the proportion of positive findings from all CTs performed after trauma. Additionally, in the second dataset, we calculated utilization as the proportion of all MHI patients who underwent CT. The second dataset was split into two periods: period one, before implementation of the new CHIP-based guideline (June 2009–September 2011) and period two, after implementation (June 2012–September 2014). The datasets contained the same months to prevent bias due to seasonal variation. Furthermore, the datasets were separated by nine months to ensure the second guideline was fully operational at the start of the period. Descriptive statistics for patient demographics and outcomes were generated. We calculated the chi-squared statistic to compare both the utilization and yield of CT between the two periods. We performed logistic regression for the effect of time on both utilization and yield during each period independently, to test whether any significant trend existed within the periods. Finally, we compared the outcomes with the results of the CHIP study in the study center. Statistical analysis was performed with R software, version 3.3.2.

## Results

### Sources and data collection

We obtained 17,237 clinical notes documented by neurology in the ED, 27,759 non-contrast head CT reports, 2088 conventional skull X-ray reports, 10,207 neurosurgical procedure registrations, 4497 hospitalizations, and 2404 records of patients who had died (i.e., irrespective of the cause of death).

### NLP performance assessment

NLP performance on 500 manually labeled clinical notes showed a 93.7% sensitivity and 97.4% specificity for the selection of acute head trauma cases, and a 97.5% sensitivity and 100% specificity for extraction of GCS score. Traumatic indication was determined with 95.8% sensitivity and 95.5% specificity on 500 manually labeled head CTs. Intracranial traumatic findings were identified with 86.8% sensitivity and 98.8% specificity on 1943 labeled head CT reports from the CHIP study. NLP errors during performance evaluation increased the tested prevalence by merely 0.25% compared to the training data.

### Dataset creation and validation

The first dataset, based only on 18,606 radiology reports from 1997 to 2009, consisted of 9109 patients with a head CT for a traumatic indication. The second dataset, based on 9153 radiology reports and 17,237 clinical reports from 2009 to 2014, consisted of 4554 MHI patients.

After inspection of 100 patients in the second dataset, we found eight patients in which the NLP algorithms identified incorrect information from the clinical records. Three were incomplete due to extraction errors (a positive scan was missed once, while an incorrect GCS was selected twice). In one patient, imaging was scheduled according to the clinical notes, but the CT report was unavailable. NLP failed to exclude two trauma patients without apparent head injury and included one patient with a previous trauma in the history. One patient was incorrectly selected after transfer from another hospital. These results are consistent with the NLP performance evaluation. Inspection of the follow-up outcomes within 30 days did not identify any misdiagnosed intracranial traumatic findings.

### Dataset 1: Historical perspective of diagnostic yield for trauma of any severity (1997–2009)

**Fig. 2:**
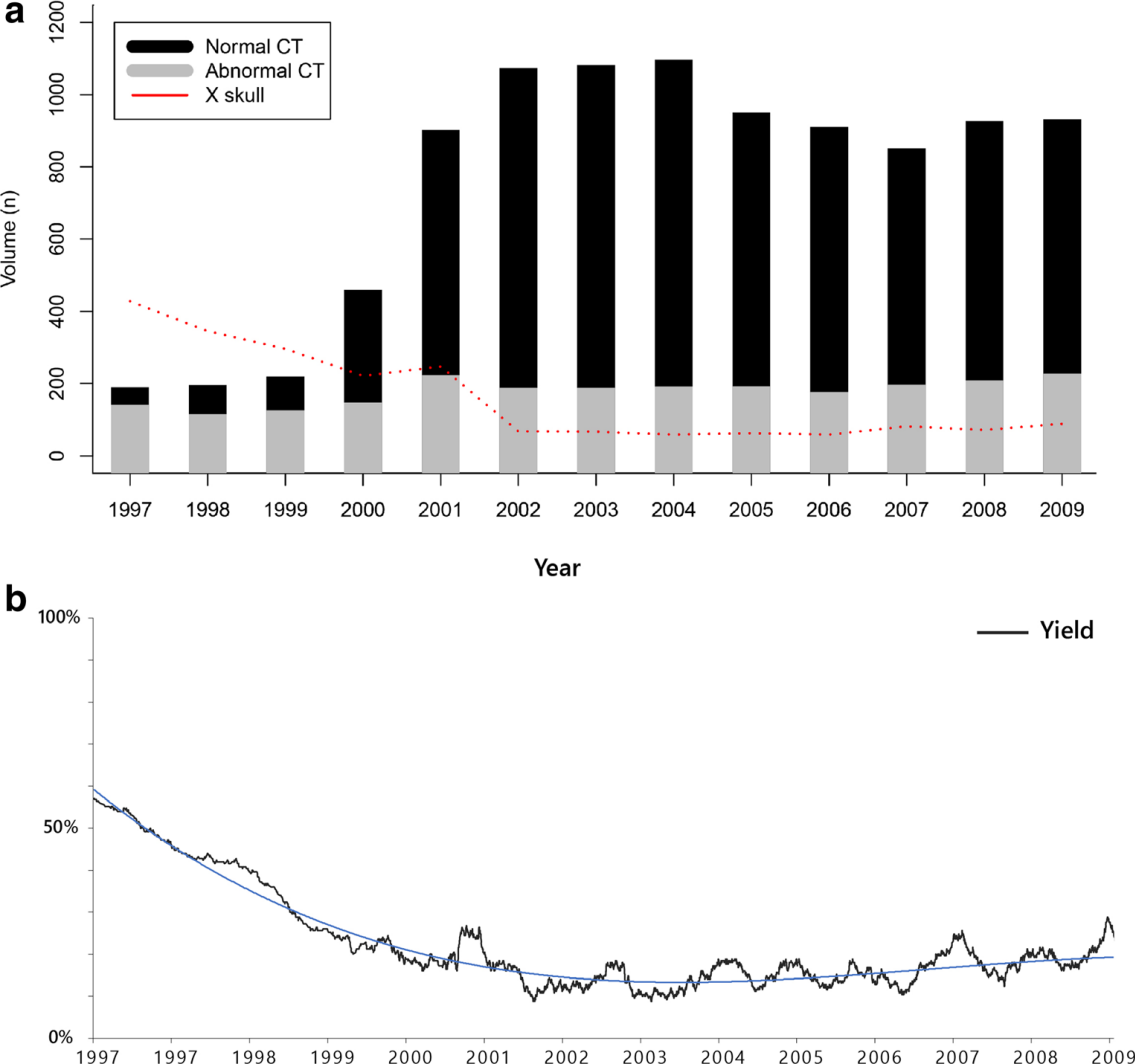
Historical perspective of CT use in patients with minor, moderate, and severe head injury from 1997 to 2009. **a** Number of patients with minor, moderate, and severe head injury and CT. The red line corresponds to skull X-ray performed for both traumatic and non-traumatic indications. **b** Yield of CT in minor, moderate, and severe head injury patients. To calculate yield for an exact point in time, we used a smoothed average of 125 entries before and after that date to calculate the proportion of positive findings

Of 9109 patients who underwent a CT after sustaining a head injury, 18.0% (*n* = 1641) had intracranial traumatic findings on CT. Over time, more CTs were performed whereas the amount of skull X-rays diminished (Fig. 2a). During the early years, a low number of CTs were performed, most of which were positive resulting in a very high diagnostic yield. From 1997, the yield was initially nearly 60%, but a decreasing trend consolidated below 20% around 2002 (Fig. 2b), which illustrates that CT had become routinely used for head trauma. The effect of the CHIP study is somewhat noticeable in the lower yield associated with scanning all patients. In subsequent years, more CTs were performed with a relatively constant number of positive findings, resulting in a lower yield (Fig. 2a, b).

### Dataset 2: Utilization and diagnostic yield of CT in MHI patients (2009–2014)

**Fig. 3:**
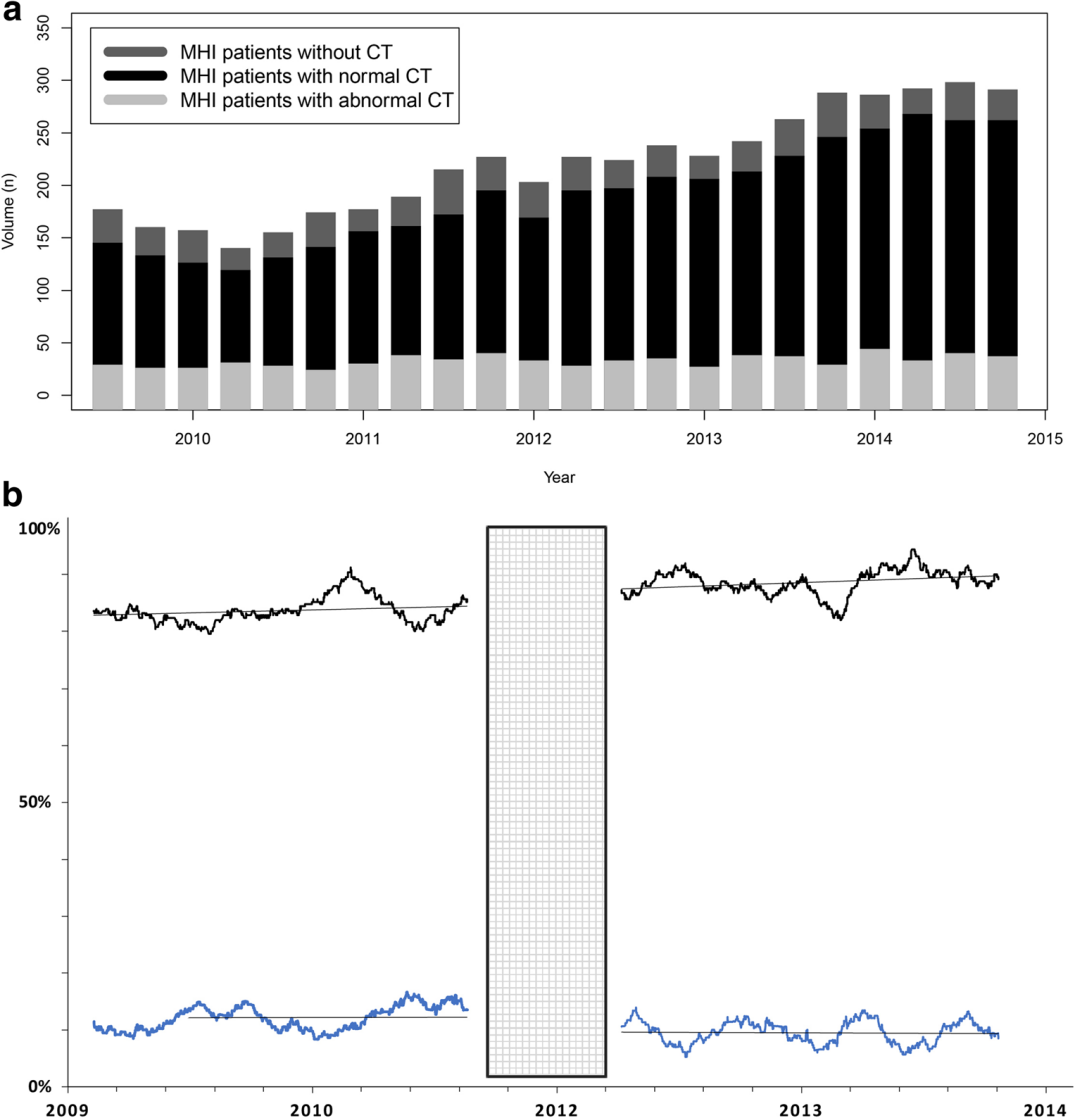
Use and yield of CT in minor head injury patients from 2009 to 2014, before and after implementation of the second guideline. **a** Number of patients with minor head injury and CT. **b** Use and yield of CT in minor head injury patients; the gray area denotes the timeframe for implementation of the second guideline. To calculate yield for an exact point in time, we used a smoothed average of 125 entries before and after that date to calculate the proportion of positive findings

For 4554 patients with MHI seen at the ED, the mean age of 45.1 (SD ± 20.3) years and most patients had GCS 15 (*n* = 3219; 70.7%) at presentation (Table 1). CT was performed in 3887 patients (85.4%), identifying 414 (9.1%) intracranial traumatic findings. Over time, the utilization of CTs in MHI increased, and the absolute number of positive findings on CT was stable, resulting in a decreasing diagnostic yield (Fig. 3a). Nine hundred seventy-seven patients (20%) were admitted to the hospital wards, and eight patients (0.18%) had a neurosurgical intervention within 30 days after injury. None of the patients without a head CT had a neurosurgical intervention within 30 days after the injury.

**Table 1:** Characteristics of patients before and after implementation of a new minor head injury guideline

	CHIP study^e^(*n* = 2193)	First period (*n* = 1429)	Second period (*n* = 2265)	Entire cohort (*n* = 4554)
Period	February 2002–August 2004	June 2009–September 2011	June 2012–September 2014	June 2009–September 2014
Mean	1575 (71.8%)	1051 (73.5%)	1536 (67.8%)	3196 (70.2%)
Mean age in years (SD)	40.3 (± 18.1)	43.4 (± 20.1)	46.5 (± 20.5)	45.1 (± 20.3)
Emergency department				
GCS 13	106 (4.8%)	109 (7.6%)	116 (5.1%)	291 (6.4%)
GCS 14	387 (17.6%)	414 (29.0%)	440 (19.4%)	1044 (22.9%)
GCS 15	1661 (75.7%)	906 (63.4%)	1709 (75.5%)	3219 (70.7%)
Use of CT^a^	2193 (100%)	1166 (81.6%)	1984 (87.6%)	3887 (85.4%)
Any intracranial traumatic finding on CT (prevalence)	155 (7.1%)	142 (9.9%)	191 (8.4%)	414 (9.1%)
Yield of CT^b^	7.1%	12.2%	9.6%	10.7%
Follow-up				
Admission to hospital^c^	–	713 (49.9%)	990 (43.7%)	2067 (45.4%)
Neurosurgical intervention (< 30 days after injury)	11 (0.50%)	3 (0.21%)	3 (0.13%)	8 (0.18%)
Death (< 30 days after presentation)^d^	–	8 (0.56%)	21 (0.93%)	33 (0.72%)

^a^Proportion of CT use in minor head injury patients. ^b^Fraction positive findings. ^c^Reason for admission to hospital unknown. ^d^Unknown cause of death. ^e^Patients with minor and at least one risk factor were included in this study. *CHIP*, CT in head injury patients; *GCS*, Glasgow Coma Scale; *ED*, emergency department; *CT*, computed tomography

### Impact of CT guidelines (second dataset)

During the CHIP study from 2002 to 2004, 2193 patients received a CT and 155 (7.1%) had intracranial traumatic findings (4). Eleven patients (0.50%) received a neurosurgical intervention within 30 days after injury.

The first guideline was used in 1429 patients from 2009 to 2011 (Table 1). Most patients were referred by ambulance (*n* = 944; 66.1%); only 26 patients were referred by a general practitioner (1.8%) and 370 patients (25.9%) came to the ED at their own initiative. In 1166 patients (81.6%), a head CT was performed and 142 (9.9%) had intracranial traumatic findings. The overall yield for the first period was 12.2%. Seven hundred thirteen patients (49.9%) were hospitalized and three of 1429 patients (0.21%) underwent a neurosurgical intervention within 30 days after injury.

The second guideline was used in 2265 patients from 2012 to 2014. One thousand five hundred two patients (66.3%) were referred by ambulance, 38 (1.7%) by a general practitioner, and 614 (27.1%) came at their own initiative. In 1984 patients (87.6%) a CT was performed, 191 patients (8.4%) had intracranial traumatic findings. The overall yield for the second period was 9.6%. Nine hundred ninety patients (43.7%) were hospitalized, and three patients (0.13%) underwent a neurosurgical intervention within 30 days after injury.

The overall increase in utilization and decrease in diagnostic yield between the two guideline periods were both statistically significant (utilization *p* = 7 × 10^−7^ and yield *p* = 0.029). Within the periods individually, we found a slightly increasing trend of yield, and during the second period, a slightly decreasing trend of yield (Fig. 3b). Both slopes were not statistically significant compared to zero (Table 2).

**Table 2:** The effect of time on use and yield of CT (2009–2014), estimated with logistic regression, where β is the increase in log odds ratio per day

	First period (June 2009–September 2011)			Second period (June 2012–September 2014)		
	Overall %	β	*p*	Overall %	β	*p*
Use	81.6	1.09 × 10^−4^	0.693	87.6	1.91 × 10^−4^	0.473
Yield	12.2	2.96 × 10^−4^	0.330	9.6	1.87 × 10^−4^	0.649

## Discussion

This study investigated the impact of clinical guidelines for the management of MHI in routine clinical practice, by assessing both utilization of CT and diagnostic yield for intracranial findings in all available electronic patient records from two decades, facilitated by NLP. After implementation of a CHIP-based guideline in 2011, the utilization of CT increased significantly, while the yield significantly decreased. Within the periods before and after the guideline change, no significant trend was found for both utilization and yield, indicating that the before-after difference can be attributed to the guideline change and is not due to a preexisting trend. Therefore, implementation of improved guidelines for selective use of CT did not reduce utilization as we expected.

Our center conducted the CHIP study from 2002 to 2004, during which all MHI patients and at least one risk factor underwent CT [4]. When all the patients were scanned, the yield is approximately equal to the prevalence. This enabled us to compare the prevalence from the CHIP study period with the yield resulting from guideline use in routine clinical practice. The yield of CT during the CHIP study was 7.1%, whereas between 2009 and 2014, selective use of CT caused a higher yield of 10.7%. The overall prevalence of intracranial traumatic findings during both guideline periods was higher (9.1%) compared to the CHIP study (7.1%). The case mix during the CHIP study may have been slightly different from patients seen by neurology at the ED in routine clinical care. Importantly, this difference is not applicable to the guideline comparison, and both percentages are in line with the known incidence of intracranial traumatic findings [3–5].

The first guideline employed in our center reduced CT utilization by 18.4% compared to scanning all patients. In 2011, the CHIP-based guideline was implemented, and the potential CT reduction compared to scanning all patients was estimated at 23–30%, but we only showed a CT reduction of 12.4%. This lower reduction might be explained by more lenient use of the CHIP criteria in routine clinical practice. While during the first guideline period, the scanning policy was effectively stricter with significantly better CT reduction; not all patients with intracranial traumatic findings were identified [8, 16, 17]. Although the risk of serious traumatic findings requiring surgery is very low, scanning all MHI patients is more cost-effective than missing only a small portion of serious traumatic findings due to selective scanning [18, 19]. Thus, if the CHIP rule facilitates detection of all serious traumatic findings, while reducing CT use by 12%, this would be preferred to scanning all patients. We have shown the effect of using a CHIP-based guideline for selective scanning in routine clinical practice, and similar results were shown in a recent external validation study with a substantial reduction in CTs in clinical practice [20]. In the hypothetical situation that CT guidelines had not been implemented, in all likelihood, all patients with at least one risk factor would be scanned similar to the CHIP study period. This would almost completely eliminate any potential CT reduction.

To evaluate the purported impact of guidelines, information about guideline adherence by clinical physicians is necessary. However, for our study period, this information was not available. Furthermore, guideline adherence may affect CT utilization; however, we have no reason to assume that implemented guidelines were treated differently in one of the periods. Previous small studies about adherence of different CT guidelines showed an adherence in 51–100% of the patients [21–24]. Guideline adherence in our center cannot be expected to be 100% for the study data, which might have led to a lower CT reduction than expected. Additionally, introduction of a new guideline may also have resulted in enhanced awareness among clinicians for the risk factors in MHI, which may have caused increased utilization of CT [25]. The purpose of guideline implementation is to optimize clinical practice and care. Therefore, guideline implementation does not necessarily lead to a decrease in imaging utilization—in fact, it may lead to an increase in imaging if previously underutilized.

Besides guideline use, other factors such as increased presentation and different referral patterns by a general practitioner or ambulance can influence CT use. We found that during the second period, the number of patients seen in the ED had increased substantially (from 2265 to 1429). This increase cannot be explained by a difference in case mix because both demographic characteristics, as well as referral patterns by general practitioners and ambulance personnel remained the same. However, the increase is in line with previous research based on national registries which identified more head injury patients presenting to the ED [11]. It is unclear whether this is caused by a potential increase in risky behavior, a higher tendency to seek urgent medical care. Other potential reasons may be an improvement of existing imaging technology, fear of litigation, or change of institutional culture, for example attitudes towards risk of missing diagnoses. Because of the increasing number of patients and the increase in the use of imaging [26, 27], efficient use of CT scanning is now more required than ever [8].

Despite, or maybe because of, scanning more patients in the second period, the number of patients admitted to the hospital was lower (49.9% vs 43.7%), which may be favorable to healthcare costs [28]. Increased CT use can lead to increased confidence among physicians that no serious injury is present and thus allows discharge from the ED without admission to the hospital. Increased use may reflect cost-conscious changes in management.

The strength of NLP enabled the extraction of large numbers of clinical variables from heterogenous EHR sources. In prior studies, NLP was used to assess diagnostic yield by extracting the imaging outcome from radiology reports [29, 30]. To our knowledge, this is the first study that investigated both the utilization and yield of diagnostic imaging by automatically extracting the indication as well as imaging outcome from free text, using multiple NLP algorithms on heterogenous EHR sources. Automatic information extraction enables the review of large numbers of textual documents but, equivalent to manual chart review, is not faultless. However, this has been shown to have limited impact, because extraction of traumatic cases and GCS was successfully optimized for very high specificity, resulting in mostly true cases in the final database. Also, during the performance evaluation, the prevalence of intracranial findings increased only by 0.25% due to errors. Any remaining errors can be assumed to affect all periods equally.

To conclude, in this large study using NLP, we showed that the number of head CTs performed for head injury gradually increased over two decades, while the diagnostic yield for intracranial traumatic findings demonstrated a decreasing trend. In 2011, despite implementation of an updated guideline aiming to improve selective use of CT for MHI, utilization significantly increased, while diagnostic yield significantly decreased. NLP is a valuable tool to monitor utilization and diagnostic yield of imaging as a potential quality-of-care indicator.

## Electronic supplementary material

ESM 1 (DOCX 18 kb)

## Abbreviations

CCHR: Canadian CT Head Rule
CHIP: CT in head injury patients
ED: Emergency departments
EHR: Electronic health record
GCS: Glasgow Coma Scale
MHI: Minor head injury
NICE: National Institute for Health and Care Excellence
NLP: Natural language processing
