# Supplementary material for: Impact of guidelines for the management of minor head injury on the utilization and diagnostic yield of CT over two decades, using natural language processing in a large dataset
Source: Eur Radiol. 2019 Jan 14;29(5):2632–40. doi: 10.1007/s00330-018-5954-5 (PMC6443919; doi:10.1007/s00330-018-5954-5)
Supplement: Supplementary file 1 — (DOCX 18 kb) [file 330_2018_5954_MOESM1_ESM.docx]

**Appendix 1. Criteria indicating the need for a CT over time**

| CHIP study | First local guideline | Second local guideline |
| --- | --- | --- |
| 2002-2004 | 2006-2011 | 2011- present time |
| CT is indicated for GCS 13-14  CT is indicated for GCS 15 and at least one risk factor:  - LOC  - Short term memory loss  - Amnesia for traumatic event  - Posttraumatic seizure  - Vomiting  - Serious headache  - Alcohol or drugs intoxication  - Injury above clavicle  - Neurologic deficit* | CT is indicated for GCS ≤14  CT is indicated for GCS 15 and at least one risk factor:  - Focal neurologic deficit ^a^  - Clinical signs of skull base fracture ^b^  - Vomiting more than once  - Retrograde amnesia > 30 minutes  CT is indicated for LOC or PTA and:  - Age ≥ 65 year  - Use of anticoagulants, coagulopathy or chronic alcohol abuse  - Dangerous trauma mechanism ^c^  - Posttraumatic seizure | CT is indicated in the presence of one major criteria:  - GCS ≤ 14  - GCS deterioration ≥ 2 points (1 hour after presentation)  - Vomiting  - Posttraumatic seizure  - Age ≥ 60 year  - Clinical signs of skull base fracture ^b^  - Dangerous trauma mechanism ^c^  - PTA ≥ 4 hours  - Use of anticoagulants, coagulopathy or chronic alcohol abuse  - Focal neurologic deficit ^a^  - Alcohol or drugs intoxication  CT is indicated in the presence of two minor criteria:  - Persistent anterograde amnesia  - Age 40-60 year  - Traumatic injury above the clavicula  - GCS deterioration with 1 point (1 hour after presentation)  - Fall from height < 1m  - PTA 2-4 hours  - LOC |

^a^ Neurologic deficit: paresis, dysphasia or other (cranial nerve damage including diplopia, changes in sensibility, asymmetrical reflexes or pathological reflexes, coordination problems and ataxia), ^b^ Clinical signs of skull base fracture: raccoon eyes, battle sign, hemotympanum, CSF otorrhea, CSF rhinorrhea, palpable discontinuity, bleeding from ear, ^c^ Dangerous trauma mechanism: pedestrian/cyclist versus vehicle, ejected from vehicle, fall from elevation (more than 1 meter or 5 stairs) or an equivalent mechanism. *CT = computed tomography, CHIP = CT in Head Injury Patients, GCS = Glasgow Coma Scale, LOC = loss of consciousness, PTA = posttraumatic amnesia*
